# Unusual presentation of eosinophilic fasciitis: two case reports and a review of the literature

**DOI:** 10.1186/1752-1947-4-46

**Published:** 2010-02-08

**Authors:** Ramazan Danis, Sami Akbulut, Abdullah Altintas, Sehmus Ozmen, Cihan Akgul Ozmen

**Affiliations:** 1 Department of Nephrology, Diyarbakir Education and Research Hospital, 21400, Diyarbakir, Turkey; 2 Department of Surgery, Diyarbakir Education and Research Hospital, 21400, Diyarbakir, Turkey; 3 Department of Hematology, Dicle University, Faculty of Medicine, 21380, Diyarbakir, Turkey; 4 Department of Radiology, Dicle University, Faculty of Medicine, 21380, Diyarbakir, Turkey

## Abstract

**Introduction:**

Eosinophilic fasciitis is an uncommon disorder with unknown etiology and a poorly understood pathogenesis. We present the cases of two patients with eosinophilic fasciitis with unusual presentation, and describe the clinical characteristics and laboratory findings related to them.

**Case presentation:**

The first case involves a 29-year-old Turkish man admitted with pain, edema and induration of his right-upper and left-lower limbs. Unilateral edema and stiffness with prominent pretibial edema was noted upon physical examination. A high eosinophil count was found on the peripheral smear. The second case involves a 63-year-old Turkish man who had pain, edema, erythema, and itching on his upper and lower extremities, which developed after strenuous physical activity. He had cervical lymphadenopathy and polyarthritis upon physical examination, and rheumatoid factor and antinuclear antibody upon laboratory examination.

**Conclusion:**

Eosinophilic fasciitis can present with various symptoms. When patients exhibit eosinophilia, arthralgia and myalgia, eosinophilic fasciitis should be considered as a possible diagnosis.

## Introduction

Eosinophilic fasciitis (EF) is an uncommon disorder with unknown etiology and a poorly understood pathogenesis. It has symmetrical involvement and in its early phase is characterized by limb or trunk erythema and edema, and later by collagenous thickening of the dermis and subcutaneous fascia. EF is a scleroderma-like syndrome that was first described in 1974 by Shulman in patients with diffuse fasciitis and eosinophilia [1-3]. This syndrome was later named EF by Rodnan *et al*. [2]. Its onset is typically acute and findings include erythema, swelling and induration of the extremities, usually accompanied by eosinophilia.

Here, we present two cases of EF with unusual presentation, and describe their corresponding clinical characteristics and laboratory findings. The first patient displayed unusual features that included high eosinophilia count and asymmetry. The second patient had cervical lymphadenopathy and polyarthritis with rheumatoid factor (RF) and antinuclear antibody (ANA).

## Case presentation

### Case report 1

A 29-year-old Turkish man was admitted to our clinic with disability because of significant pain, edema and stiffness of his right-upper and left-lower limbs. He reported that the same clinical picture first appeared 3 years prior to this presentation and had since been repeated many times. His condition sometimes improved spontaneously and other times with the use of non-steroidal anti-inflammatory drugs (NSAIDs). Unilateral edema and stiffness in his right-upper limb (left arm circumference was 28.5 cm and right arm circumference was 30.5 cm) and left-lower limb (left thigh circumference was 53 cm and right thigh circumference was 46.4 cm), with prominent non-pitting pretibial edema were detected upon physical examination. His white blood cell count (WBC) was 22.8 × 10^9^/L with 26.4% neutrophils, 11.2% lymphocytes, and 60% eosinophils. His hemoglobin was 14.6 gdL, and his erythrocyte sedimentation rate (ESR) was 3 mm/h.

**Figure 1:**
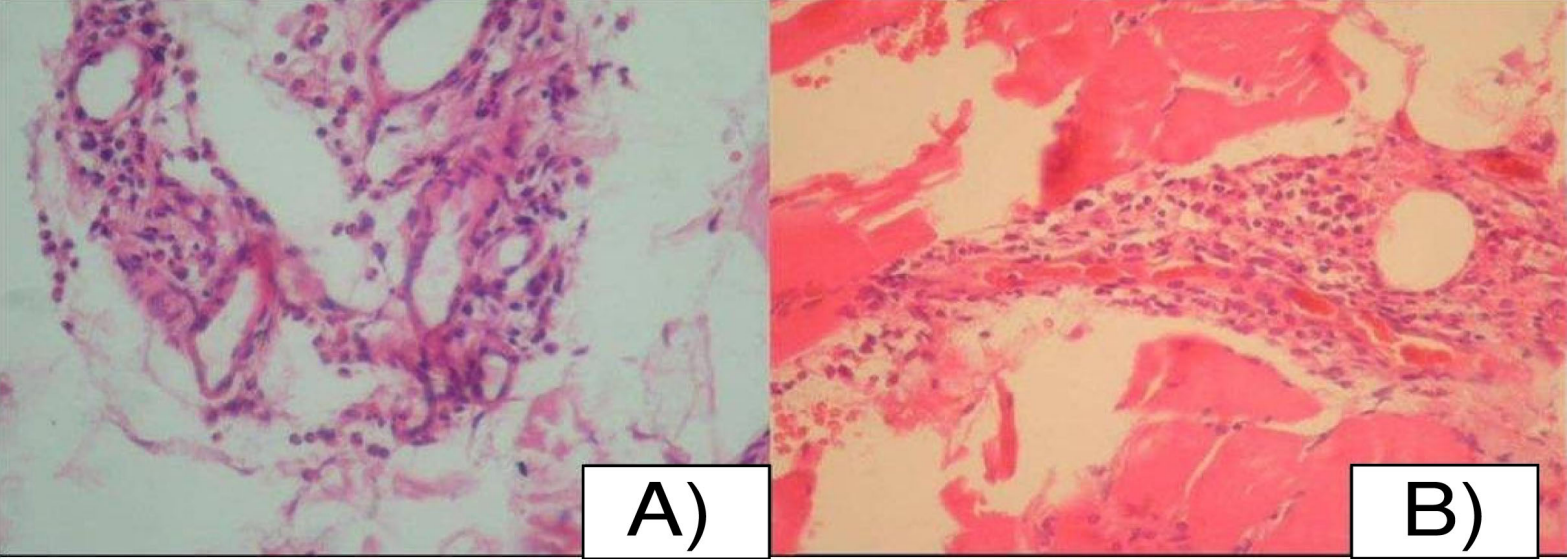
**Mixed-type infiltration of eosinophils and other inflammatory cells in muscle and fat tissues of (A) patient 1 and (B) patient 2**. Hematoxylin and eosin stain, magnification ×200.

**Figure 2:**
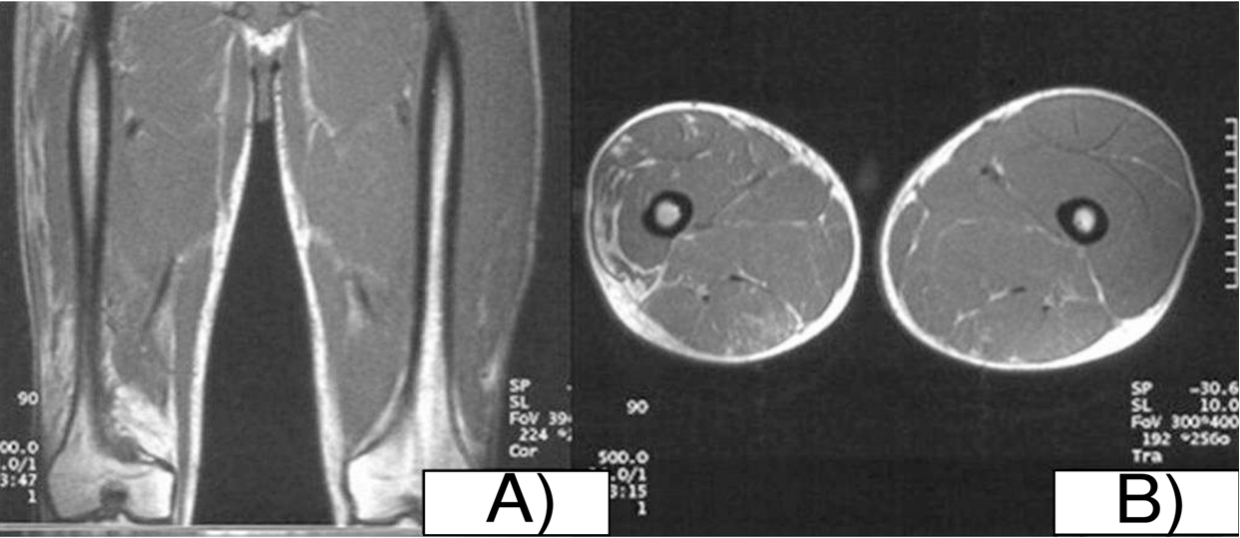
**Coronal and axial magnetic resonance imaging of patient 1**. His left extremity was thicker than his right extremity, as shown on coronal (A) and axial (B) images.

Our patient's stool specimens were examined for ova and parasites. Meanwhile, his renal, thyroid and liver function tests yielded negative results. His electrolytes were also within normal limits. Results were also negative for RF, C-reactive protein and ANA. Results of his chest radiography, esophagography, abdominal ultrasonography and pulmonary-function studies were all within normal limits. Bone marrow aspirate smears showed 60% eosinophils. A full-thickness biopsy of his left calf revealed active fasciitis (Figure 1A). Magnetic resonance imaging of his lower limbs revealed that his left-limb muscle group was thicker than his right (Figures 2A and 2B).

Finally, a diagnosis of EF was established from these clinical and laboratory findings. His symptoms disappeared completely after a few days of treatment with 1 mg/kg/day oral methylprednisolone.

### Case report 2

A 63-year-old Turkish man was admitted to our clinic with edema, erythema, pain and itching of his upper and lower extremities for 10 days, which started after strenuous physical activity working with an axe in the forest. Mobile, palpable lymph nodes were found in the right anterior cervical (2 × 1 cm), left submandibular (3 × 1 cm) and left submental (2 × 2 cm) regions of his body. His shoulder and elbow joints were warm, while their range of movement, as well as the flexion and extension of his wrist, were limited. Both knee joints were warm and painful on flexion. His WBC count was 12.9 × 10^9^/L, while his neutrophils was 5.3 × 10^9^/L, eosinophils was 4.9 × 10^9^/L (37.9%), and ESR was 98 mm/h. His ANA was positive, and his RF was 0.59 IU/L. Peripheral blood smears showed 34% eosinophils. An examination of his stool specimens returned negative for ova and parasites. His electrolytes, renal, thyroid and liver function values were all within normal limits. Results of his chest radiography, abdominal ultrasonography, and pulmonary-function studies were also within normal limits. Mild hepatomegaly (165 mm) was detected upon abdominal ultrasonography. A full-thickness biopsy revealed active fasciitis (Figure 1B). A diagnosis of EF was established from these clinical and laboratory findings. His symptoms improved completely after a few days of treatment with 1 mg/kg/day oral methylprednisolone.

## Discussion

EF is an uncommon disease and only a few hundred cases have been reported in the literature. It is characterized by acute or subacute symmetric swelling of the skin and the subcutaneous tissues. The forearms, flanks and upper legs are usually affected, while the hands and face are spared [4]. However, our first patient had asymmetric edema and pain in his right limb, shoulder and face, which differed from other cases reported in the literature.

While the etiology of EF is still unknown, possible causes include strenuous exercise, initiation of hemodialysis, and infection with *Borrelia burgdorferi *[1,5,6]. In addition, exposure to some drugs has been implicated. Cutaneous side effects following simvastatin treatment, including the development of EF, have been well-documented [7].

None of these causes were obvious in the first case we presented, but strenuous exercise appeared to be the triggering factor for the second patient. There was no suspicion of relevant environmental or toxic exposure in either of our patients. Paraneoplastic disease, progressive systemic sclerosis, and infection with *B. burgdorferi *were thus excluded.

The majority of patients with EF have peripheral blood eosinophilia during the acute phase of the disease. In one series, 33 out of 52 patients had eosinophilia. Elevated ESR (29%) and polyclonal hypergammaglobulinemia (35%) can also be found [8]. ANA positivity has not been reported previously in EF with any consistency [3], and RF is almost always negative. Both our patients had hypereosinophilia, and our second patient had an increased RF (0.59 IU/L) and a positive ANA test. Definitive diagnosis requires histopathological examination from a full-thickness (epidermis to muscle) biopsy [9]. The biopsy results of both patients were consistent for EF upon histopathological examination.

There is substantial agreement among published cases or case series that corticosteroids are the first-line treatment for EF and are usually effective in >70% of cases. Other treatments include NSAIDs, D-penicillamine, chloroquine, cimetidine, methotrexate, azathioprine, cyclosporin A, infliximab, UVA-1, and bath PUVA [10,11].

Spontaneous remission rate in patients with EF is 10% to 20% at the time of presentation or relapse after discontinuing corticosteroid therapy [12]. Our first patient had a history of spontaneous remission. In one series, hematological disorders other than eosinophilia were present in 5 out of 52 patients [8]. Hematological abnormalities that have been described in association with EF include aplastic anemia, acquired amegakaryocytic thrombocytopenia, myeloproliferative disorders, myelodysplastic syndromes, lymphoma, leukemia, and multiple myeloma [8]. However, there was no hematological abnormality in our patients we described.

The presence of lymphadenopathy is unusual. Ten reported cases of EF with enlarged lymph nodes have been identified previously. Six of these patients had lymphoma and four had reactive lymphadenopathy [13]. Our second patient had cervical, submandibular and submental mobile lymphadenopathy, with an enlarged liver and no haematological disease.

Two cases of EF with rheumatoid arthritis (RA) have been reported previously, but the diagnosis of RA had been established in these patients before the diagnosis of EF [14,15]. In the second case we described, our patient's symptoms at first were like those of RA. However, the symptoms began shortly after strenuous exercise, which is not typical for RA, and eosinophilia and histopathological evaluation revealed the correct diagnosis. Furthermore, the symptoms did not meet RA criteria. Most EF patients with arthritis complain of morning stiffness and exhibit changes on joint radiographs similar to patients with RA [8]. This condition may thus lead to misdiagnosis.

Magnetic resonance imaging (MRI) can be used for the diagnosis of EF [9,15,16]. In two retrospective studies involving seven patients, MRI detected fascial thickening and signal abnormalities in patients with EF at the time of diagnosis [9,15]. MRI showed evidence of disease activity in both of our patients.

## Conclusions

EF can present with various symptoms. When patients exhibit eosinophilia, arthralgia and myalgia, EF should be considered as a possible diagnosis. It is notable that the first patient described in this case report also displayed unusual features including high eosinophil count and asymmetrical presentation.

## Abbreviations

EF: Eosinophilic fasciitis; RA: Rheumatoid arthritis; ANA: Antinuclear antibody; ESR: Erythrocyte sedimentation rate; MRI: Magnetic resonance imaging.

## Consent

Written informed consent was obtained from our patients for publication of this case report and any accompanying image. Copies of the written consent are available for review by the Editor-in-Chief of this journal.

## Competing interests

The authors declare that they have no competing interests.

## Authors' contributions

RD, SA, AA and SO contributed in writing the manuscript and in reviewing the literature. SA, RD and AA contributed in this case report's design and in preparing the manuscript for publication. CAO provided the necessary radiological information. All authors read and approved the final manuscript.
